# Identification and Expression Analysis of Aquaporins in the Potato Psyllid, Bactericera cockerelli

**DOI:** 10.1371/journal.pone.0111745

**Published:** 2014-10-29

**Authors:** Freddy Ibanez, Joseph Hancock, Cecilia Tamborindeguy

**Affiliations:** Department of Entomology, Texas A&M University, College station, Texas, United States of America; Volcani Center, Israel

## Abstract

Aquaporin (AQPs) proteins transport water and uncharged low molecular-weight solutes across biological membranes. Six to 8 AQP genes have been identified in many insect species, but presently only three aquaporins have been characterized in phloem feeding insects. The objective of this study was to identify candidate AQPs in the potato psyllid, *Bactericera cockerelli*. Herein, we identified four candidate aquaporin cDNAs in *B. cockerelli* transcriptome. Phylogenetic analysis showed that candidate BcAQP2-like had high similarity to PRIP aquaporins; while candidates BcAQP4-like, BcAQP5-like and BcAQP9-like clustered within clade B. In particular, candidates BcAQP4-like and BcAQP5-like clustered with functionally validated insect aquaglyceroporin proteins. Expression analyses using RT-qPCR showed that all candidates were expressed in all life stages and tissues. Candidates BcAQP4-like and BcAQP5-like were highly expressed in bacteriocytes, while BcAQP9-like appeared to be expressed at high levels in whole body but not in the assayed tissues. This study is the first global attempt to identify putative aquaporins in a phloem feeding insect.

## Introduction

Plant sap feeders from the order Hemiptera are important agricultural pests; they can damage crops directly through their feeding and excretion activities on host plants or indirectly as vectors of plant pathogens [1]. As any other living organism, these insects need to exert a fine regulation of water movement within their bodies to avoid desiccation. Phloem feeding hemipterans face an additional challenge, their diet is exclusively liquid but phloem’s osmotic pressure is two to five times higher than the insect’s hemolymph [2]. Their success is believed to rely on two biological adaptations to lower the osmotic pressure of the ingested sap. First, it is hypothesized that sucrose from the phloem is hydrolyzed and the resulting glucose is converted into oligosaccharides and excreted as shown in *Acyrthosiphon pisum* [2], [3], [4], [5], while in *Bemisia argentifolii* under high sucrose concentration, sucrose is isomerized into trehalulose and excreted [6]. Second, water is hypothesized to cycle within the gut. In aphids, this is allowed by the gut structure, which often forms a loop bringing proximal and distal regions to proximity [7], while in other hemipterans such as whiteflies and psyllids water cycling occurs within the filter chamber [8], [9]. Aquaporins are believed to play a central role in water cycling within the gut of hemipterans [7], [8].

Aquaporins (AQP) are proteins involved in water transport across membranes. Since their identification in 1980’s in humans [10], [11], aquaporins have been found in plants, animals and microorganisms [12], [13], [14]. In mammals, 13 AQPs have been identified. Based on differences in structure and solute selectivity, mammalian AQPs are classified in three subfamilies. The traditional (Class 1) aquaporins are mostly water selective (AQP0, 1, 2, 4, 5, 6 and 8) though some are not water selective and transport anions (AQP6) or free radicals (AQP8) [15], [16]. The aquaporins in the second subfamily (Class 2) are called aquaglyceroporins since they also transport small neutral solutes like urea, glycerol and metalloids (AQP3, 7, 9 and 10) [17]. The aquaporins (AQP11 and 12) in the third subfamily (Class 3), are called superaquaporins and are classified together based on the presence of only 1 NPA motif and a variant motif, NPT for AQP12 [18] and NPC for AQP11 [19]. Despite extensive studies on mammalian AQPs, studies on insect AQPs are not as extensive. Putative AQP homologues have been found in a wide range of insect genomic databases [20] and 6 to 8 AQP genes have been identified in many insect species. Recent studies have identified aquaporins in hemipterans [21], [22]. However, presently only three aquaporins from phloem feeding hemipterans have been characterized. Two of them, *A. pisum* ApAQP1 and *Bemisia tabaci* BtAQP1, belong to the insect DRIP family and are expressed in the insect gut [7], [8]. The third gene, *A. pisum* ApAQP2, is expressed in the insect bacteriocyte and fat body and appears to be an aquaglyceroporin [23].

Our aims for this study were to identify putative AQP genes in the potato psyllid *Bactericera cockerelli* (Sulc) (Hemiptera: Triozidae), a phloem feeding insect, using the published transcriptome resource [24], to evaluate their expression pattern in different life stages, and localize their expression in adult tissues using *in situ* hybridization assays.

## Materials and Methods

### Insects

*Bactericera cockerelli* colonies were maintained on tomato plants in 14″ X 14″ X 24″ insect cages (BioQuip, Rancho Dominguez, CA, USA) at room temperature and photoperiod of 16∶8 h (light:dark).

### Sequence identification and cloning

*Bactericera cockerelli* transcriptomic dataset [24] was datamined to identify putative aquaporin sequences using BLASTX. Specific primers were designed using Primer 3 software for each candidate aquaporin (Table 1). Total RNA from twenty adult *B. cockerelli* was extracted using Trizol reagent (Life Technologies, Carlsbad, CA) following the manufacturer’s instructions. Genomic DNA contamination was eliminated by DNase treatment with Turbo RNase-free DNase (Life Technologies, Carlsbad, CA). The total RNA quantity and purity were determined using a Biophotometer plus (Eppendorf, Hamburg, Germany) and RNA integrity was visualized by electrophoresis in 1.2% agarose gels stained with ethidium bromide. Five hundred ng of total RNA were processed for cDNA synthesis using Verso cDNA Synthesis kit (Thermo, Waltham, MA) with Anchored Oligo(dT) primers following the manufacturer’s instructions. The candidate aquaporins were amplified by PCR using specific primers for each gene and PrimeSTAR Max DNA Polymerase (Clontech, Mountain View, CA) according to the manufacturer’s recommendations. Each reaction contained 25 ng of cDNA, 150 nM of each primer and 1X of PrimeSTAR Max Premix, the volume was adjusted with nuclease-free water to 50 µL. The PCR conditions were: 95°C for 2 min; followed by 30 cycles of 95°C for 30 sec, 60°C for 30 sec, and 72°C for 1 min; and a final extension at 72°C for 3 min. PCR products were examined by gel electrophoresis, purified using PureLink Quick Gel Extraction kit (Life Technologies, Carlsbad, CA), and cloned into the pGEM-T vector using the pGEM-T Easy cloning kit (Promega, Madison, WI) following the manufacturer’s recommendations. Plasmids were purified using PureLink Quick Plasmid Miniprep Kit (Life Technologies, Carlsbad, CA) and sequenced by Eton bioscience Inc.

**Table 1:** Primers used to amplify full length *B. cockerelli* aquaporin candidates and to produce probes for *in situ* hybridizations.

Name	Sequence	Amplicon size (bp)
**Primers used to amplify full length CDS**		
BcAQP2-likeFL-F	5-GTTTCGGACTTTGCGAATTT-3	1,012
BcAQP2-likeFL-R	5-TGAAATTTTCGAGAGGCAGT-3	
BcAQP4-likeFL-F	5-AAACAAGAGTATCGCCAAGAGC-3	941
BcAQP4-likeFL-R	5-TCCATTATTGTTAGCATTTGTTTT-3	
BcAQP5-likeFL-F	5-AAAAGCTAGGGAAATACTTCGACA-3	1,002
BcAQP5-likeFL-R	5-TGAAGACACAGATTTCAACACAGA-3	
BcAQP9-likeFL-F	5-TGACCAGCTGGACCTATTACC-3	1,110
BcAQP9-likeFL-R	5-TGATTTTCCTTCTCATGCACA-3	
**Primers used to produce ** ***in situ*** ** hybridization probes**		
BcAQP2-likeHyb-F	5-TTTCGGGTGTCTGAGTTGTG-3	614
BcAQP2-likeHyb-R	5-GCGCCACAAAGAAGAATGTG-3	
BcAQP4-likeHyb-F	5-CGAGAGGTTTCTCCACATATCC-3	617
BcAQP4-likeHyb-R	5-GAGCCACCCAGTAGATCCAA-3	
BcAQP5-likeHyb-F	5-TCCTCCCTAGGTCACAAGAAA-3	642
BcAQP5-likeHyb-R	5-AGTCCACACCTGGGAGAATA-3	
BcAQP9-likeHyb-F	5-AAACAAGAGTATCGCCAAGAGC-3	421
BcAQP9-likeHyb-R	5-AGCCAGCACATACACCACAG-3	

### Dissection of Tissues

At least twenty-four adult *B. cockerelli* per replicate (N = 3) were randomly picked from the laboratory colony and placed in Eppendorf tubes on ice until dissection. Guts, bacteriocytes, female and male reproductive organs were dissected using a dissection slide treated with RNase Zap (Life Technologies, Carlsbad, CA) in 0.9% (w/v) NaCl solution. The tissues were placed in Trizol reagent (Life Technologies, Carlsbad, CA) for total RNA extraction or 1x Phosphate-buffered Saline (PBS) for *in situ* hybridizations.

### Life stages

Fifty eggs, 20 4^th^–5th instar nymphs, 20 2–3 day old adults (10 females and 10 males), and 20 7-day old adults (10 females and 10 males) per replicate (N = 3) were picked from the laboratory colony and placed in Trizol reagent (Life Technologies, Carlsbad, CA) for total RNA extraction.

### Expression analyses by RT-qPCR

Total RNA from different tissues (100 guts, bacteriocytes, female and male reproductive organs) of adult *B. cockerelli*, as well as from different life stages (eggs, 4th–5th instar nymphs, 2–3 day old female and male adults, and 7-day old female and male adults) were extracted using Trizol reagent (Life Technologies, Carlsbad, CA) following the manufacturer’s instructions and cDNA was synthetized as previously described. Two independent replicates for each dissected tissue were performed.

For each aquaporin candidate, RT-qPCR reactions were performed using SensiFAST SYBR Hi-ROX Kit (Bioline, Taunton, MA) according to manufacturer’s instructions. Each reaction contained 5 ng of cDNA, 250 nM of each primer and 1X of SYBR Green Master Mix; the volume was adjusted with nuclease-free water to 10 µL. The real-time PCR program was 95°C for 2 min followed by 40 cycles at 95°C for 5 sec and 60°C for 30 sec. Primers were designed using Primer3 web [25] (Table 2). Real-time PCR assays were performed using an Applied Biosystems ABI 7300 real-time PCR Thermocycler (Applied Biosystems) according to manufacturer’s recommendations. Reactions for all samples were performed in duplicates with a negative control in each run. The threshold cycle (Ct) values and the efficiency of each primer set for RT-qPCR were determined using LinRegPCR software [26]. The relative expression of each aquaporin candidate was estimated by normalizing transcript levels of genes of interest to the internal control gene *B. cockerelli* Ribosomal protein S18 (RPS18) (Ibanez and Tamborindeguy, unpublished) expression values. In each comparison, the candidate with the lowest 2^−ΔCT^ value was used as calibrator with a value of “1”.

**Table 2:** Primers used for RT-qPCR analyses of *B. cockerelli* aquaporin candidates.

Name	Sequence	Efficiency
BcAQP2-likeqP-F	5-TTTCGGGTGTCTGAGTTGTG-3	96.7
BcAQP2-likeqP-R	5-GTGAAGATGACGAGACCGAAC-3	
BcAQP4-likeqP-F	5-TAATCCAGCGGGTAACAACC-3	95.9
BcAQP4-likeqP-R	5-AAGGAGGTTCCCAAAAGCTC-3	
BcAQP5-likeqP-F	5-AGCTTCCAGTGTATTTCGTCTC-3	95.8
BcAQP5-likeqP-R	5-GGATCTCTTGAGGCGTGACC-3	
BcAQP9-likeqP-F	5-TGGAGTGTGGGATAATCATTG-3	96.2
BcAQP9-likeqP-R	5-TGCTTGTCTGGATTGTCGTAG-3	
BcRPS18-qP-F	5-GCGAGTGTTTGGTCGTCATA-3	97.0
BcRPS18-qP-R	5-TGACTGGCGGGCTTTTATTA-3	

### *In situ* hybridization analyses

Aquaporin candidate PCR products for *in situ* hybridizations were obtained using specific primers (Table 1) and PrimeSTAR Max DNA Polymerase (Clontech, Mountain View, CA), following the manufacturer’s recommendations and PCR conditions previously mentioned. Amplicon sizes were 614, 617, 642 and 421 bp for BcAQP2-like, BcAQP4-like, BcAQP5-like, and BcAQP9-like, respectively. These amplicons were cloned into pGEM T Easy vector (Promega, Madison, WI) and sequenced to determine insert orientation. Plasmids with aquaporin inserts were purified using PureLink Quick Plasmid Miniprep Kit (Life Technologies, Carlsbad, CA) and linearized by specific restriction enzymes during 8 hours at 37°C. The degree of linearization was examined on a 1% agarose gel. After complete digestion, linearized plasmids were cleaned by ethanol precipitation (0.3 M sodium acetate, pH 5.2; 75% ethanol, placed at −20°C for at least 120 min) and centrifuged at 12,000 rpm for 30 min at 4°C. Subsequently, 1 µg of linearized plasmids were used to synthetize the sense and antisense RNA probes by *in vitro* transcription using DIG RNA labeling kit (Roche, Penzberg, Germany) following the manufacturer’s instructions.

Guts, bacteriocytes, female and male reproductive organs were dissected from adult *B. cockerelli* as previously described then fixed in 3.8% formaldehyde in 1x PBS at room temperature during 2 hours. The tissues were washed once for 5 min with 1x Phosphate-buffered saline, 0.1% (v/v) Tween 20 (PBST) and dehydrated with 100% methanol at −20°C until further processing. The tissues were rehydrated through a graded series of methanol/PBST, washed 3 times for 5 min in PBST. The hybridization was carried out overnight at 60°C with 2.0 ng/µL sense or antisense DIG-dUTP labeled RNA probes in hybridization solution (50% (v/v) formamide, 5x SSC, 1 mg/mL total yeast RNA, 100 mg/mL heparin, 0.1% (v/v) Tween 20). After hybridization, unbound probes were washed off at 60°C with the following steps: 2x SSC, 1 hour; and 0.2x SSC twice, 1 hour each. After, the tissues were washed gently twice with maleic acid buffer (100 mM maleic acid, 150 mM NaCl, 0.1% (v/v) Tween 20 pH 7.5) at room temperature for 10 min, and blocked in 1x Blocking Reagent (Roche, Penzberg, Germany) for 2.5 hours at room temperature. The tissues were then incubated with anti-DIG AP fragments antibody (Roche, Penzberg, Germany) at 1∶2000 in 1x blocking solution with gently shaking (50 rpm) overnight at 4°C. The antibody was detected after four washes for 20 min at room temperature in maleic acid buffer and color was developed using BM Purple alkaline phosphatase substrate (Roche, Penzberg, Germany) plus 5 mM levamisole to block or avoid endogenous alkaline phosphatase activity. *In situ* images were obtained with an Axioimager A1 microscope (Carl Zeiss microimaging, Thornwood, NY, USA) and visualized with Axiovision Rel 4.8 software (Carl Zeiss).

### Phylogenetic and bioinformatic analyses

Candidate aquaporin sequences were *in silico* translated using Expasy Translate tool [27]. Topology prediction of transmembrane helices was performed using TMHMM program [28].

The predicted amino acid sequences were aligned with 58 animal aquaporins. Aquaporin sequences were truncated to remove the predicted N and C terminal cytoplasmic tails, which were variable and could not be aligned with confidence for phylogenetic analysis. Alignment was performed using ClustalW [29] and phylogenetic linkage of the protein sequences was assessed by Bayesian inference ran in MrBayes 3.2 software [30] with the following parameters: four chains, two runs, amino acid model = GTR (Wag), rate variation = “invgamma” (GTR+I+Gamma model), and the analysis of Metropolis-coupled Markov chain Monte Carlo (MCMC) run was four million generations, sampled every 1000th step, and the first 25% of sampled trees were discarded as burn-in. The runs were considered converged when average standard deviation was lower than 0.01 and potential scale reduction factor value (PSRF) approached 1.0. The values of branch support were obtained by the method of posterior probability (≥0.70). The tree was rooted at midpoint, and saved and edited by Figtree program v.1.4.0.

## Results

### Identification of *Bactericera cockerelli* aquaporins

Datamining of *B. cockerelli* transcriptome resources [24] resulted in the identification of four putative full-length aquaporin candidates based on sequence similarities to other described insect aquaporins [7], [23], [31]. For each candidate, the putative full length CDS was amplified, cloned and sequenced. The start codon for each candidate was determined by analysis of the sequence upstream ATG to find the motif that conformed more closely to the optimal Kozak motif [32]. Candidate aquaporins were named based on their similarity to other insect aquaporins: BcAQP2-like, BcAQP4-like, BcAQP5-like and BcAQP9-like, GenBank KF649616, KF649617, KF649618 and KF649619; and they were predicted to encode proteins of 266, 260, 279 and 269 amino acids, respectively.

**Figure 1:**
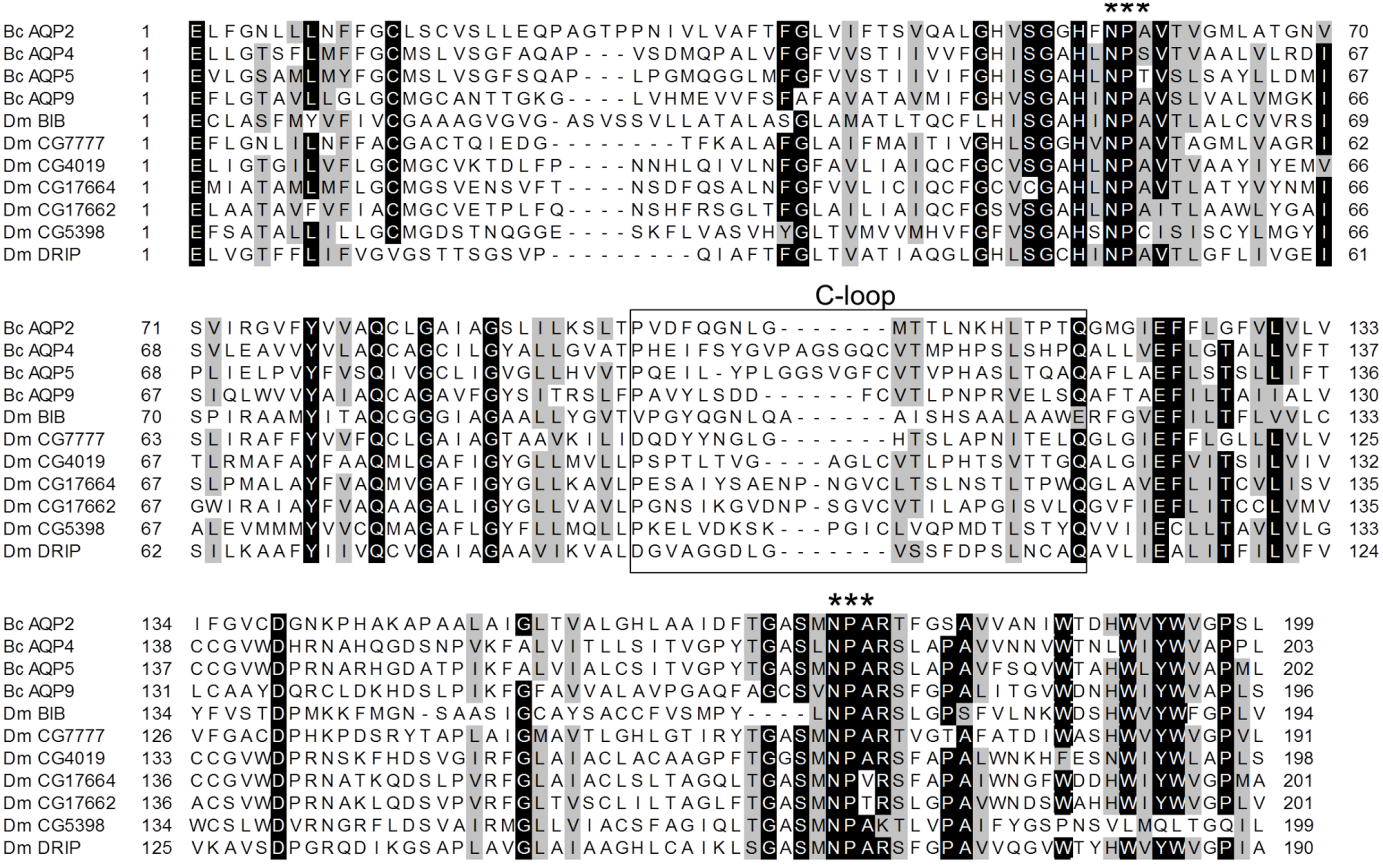
Amino acid sequence alignment of *B. cockerelli* and *D. melanogaster* putative aquaporins. NH2- and COOH-terminal regions are not included. Fully conserved amino acids are shaded in black, whereas conserved substitutions are shaded in grey. NPA motifs are highlighted with asterisks (*). Box represents C-loop. Longer C-loop might suggest an aquaglyceroporin function [31].

Bioinformatic analyses using TMHMM program identified six predicted transmembrane regions within all aquaporin candidate protein sequences. Two of the candidates, BcAQP2-like and BcAQP9-like contained both NPA motifs, whereas BcAQP4-like and BcAQP5-like contained a NPS and a NPT instead of the first NPA motif, respectively (Figure 1 and Figures S1–S4).

**Figure 2:**
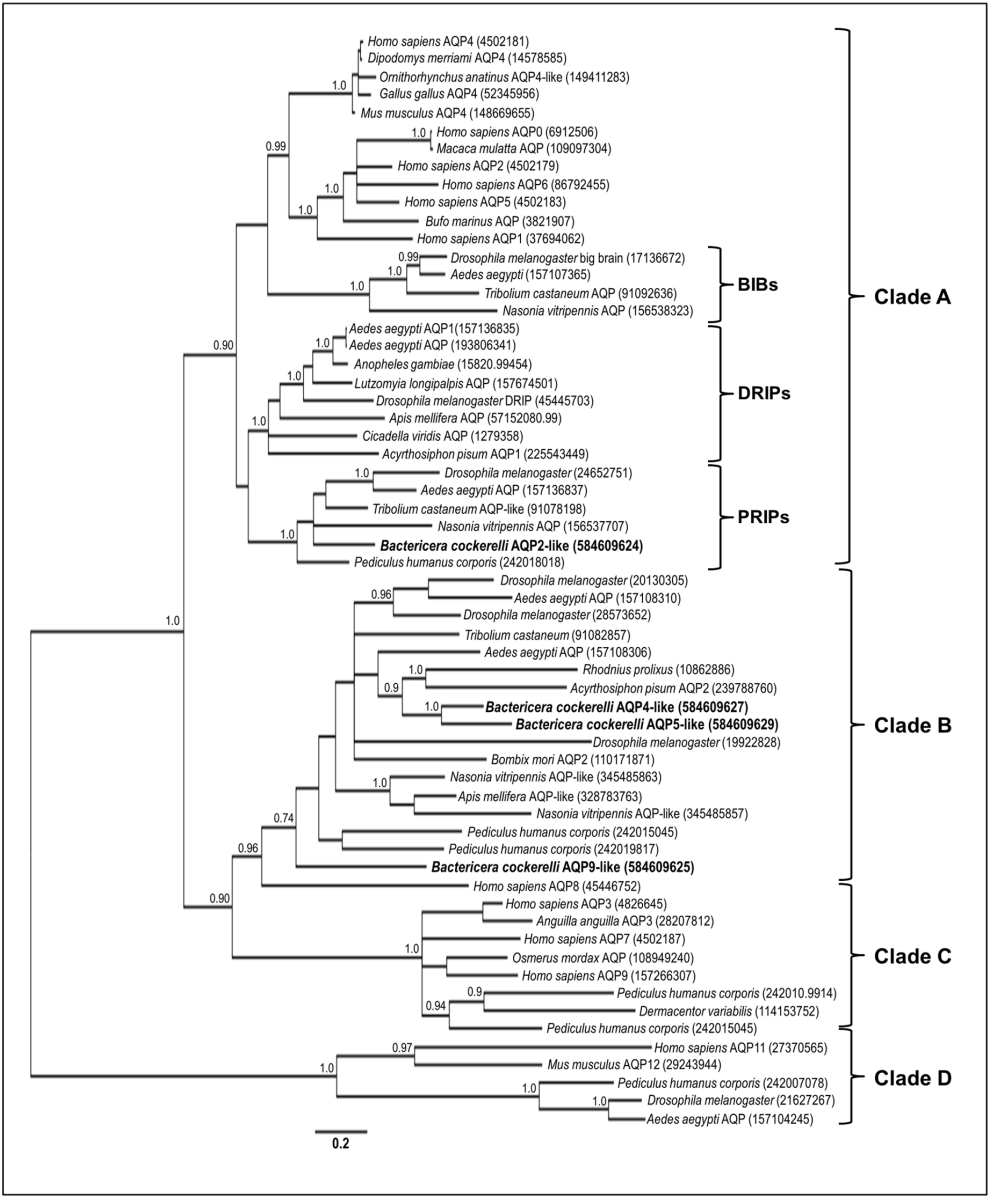
Phylogenetic analysis of *B. cockerelli* aquaporin candidates and other animal aquaporins under Bayesian inference. The numbers at the nodes denote posterior probabilities, only values higher than 0.70 are shown. The four phylogenetically supported AQP clades (A–D) previously described [23] are shown.

A Bayesian analysis was conducted to evaluate the association among the deduced protein sequences with other animal aquaporins (Figure 2). The deduced BcAQP2-like amino acid sequence clustered within clade A and in the same integral protein subfamily as *D. melanogaster* CG7777 and *A. aegypti* AaeAQP2, both classified as PRIP aquaporins. None of the *B. cockerelli* candidates clustered within the integral protein subfamilies DRIP or BIB.

The deduced amino acid sequences of BcAQP4-like and BcAQP5-like clustered within clade B, previously described as an insect-specific aquaporin clade [22], [23], [33]. These two *B. cockerelli* candidates clustered within a node composed by several aquaglyceroporins such as ApAQP2 [23] or *Bombix mori* AQP2 [34]. Candidates BcAQP4-like and BcAQP5-like had a longer C-loop region according to sequence alignment performed with several *D. melanogaster* aquaporin protein sequences (Figure 1). This longer C-loop region might suggest an aquaglyceroporin function [31].

BcAQP9-like was clustered within clade B. However, this candidate protein seemed to be more divergent than the other insect aquaporins in this clade. More aquaporin sequences are needed to obtain a better resolution of this clade.

### Expression of *Bactericera cockerelli* aquaporins in adults

**Figure 3:**
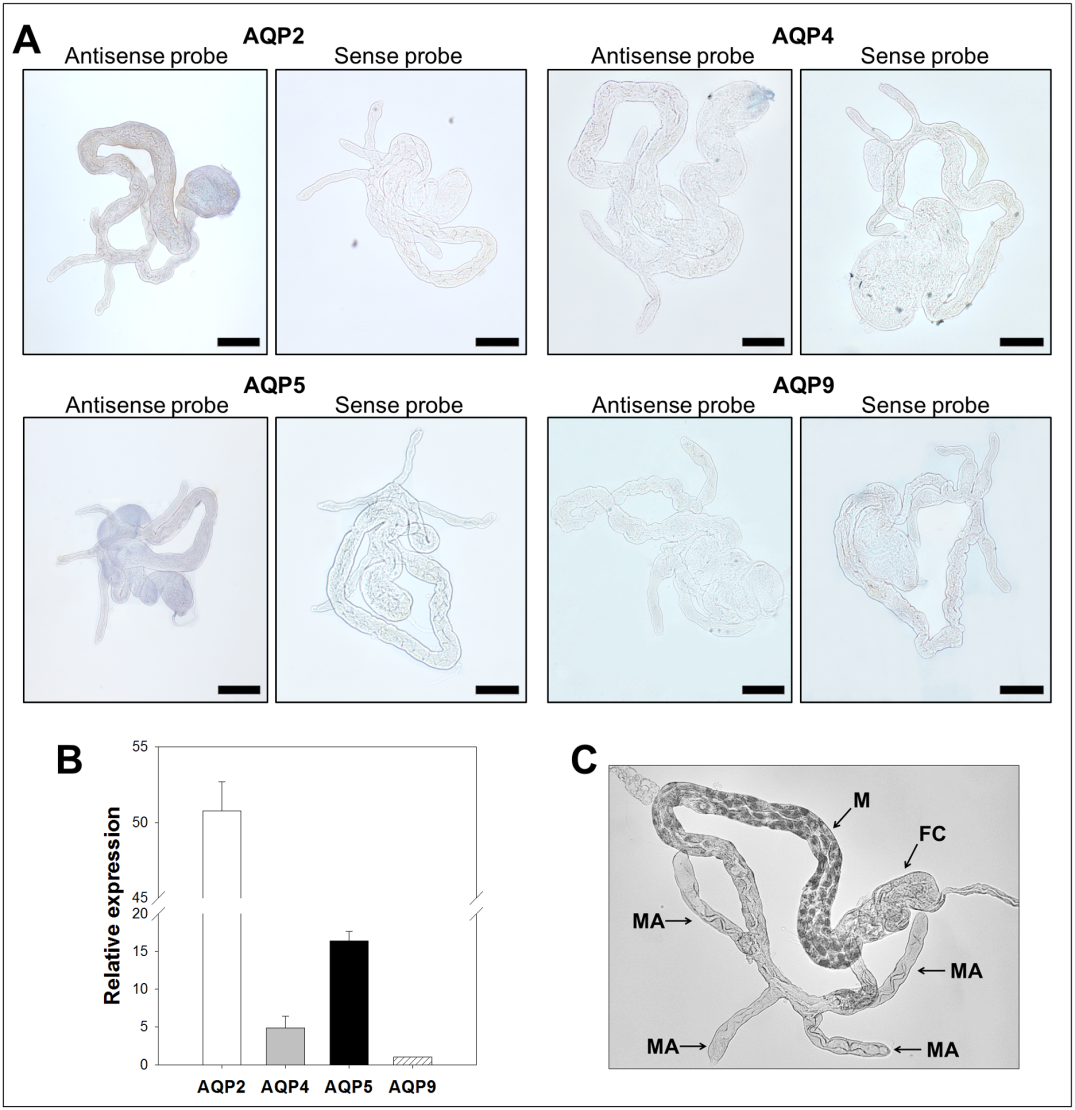
Expression analysis of *B. cockerelli* aquaporin candidates in guts. A: *In situ* hybridization of candidate mRNAs with digoxigenin-labelled antisense and sense RNA probes (original magnification: ×5, bars = 200 µm). B: Relative gene expression levels of *B. cockerelli* aquaporin candidate genes normalized to the expression value of the RPS18 gene. Data points represent means ± SD of two independent experiments performed in duplicates. C. Light micrograph of *B. cockerelli* alimentary canal showing characteristic structures. FC: filter chamber, M: midgut, MA: midgut appendages.

**Figure 4:**
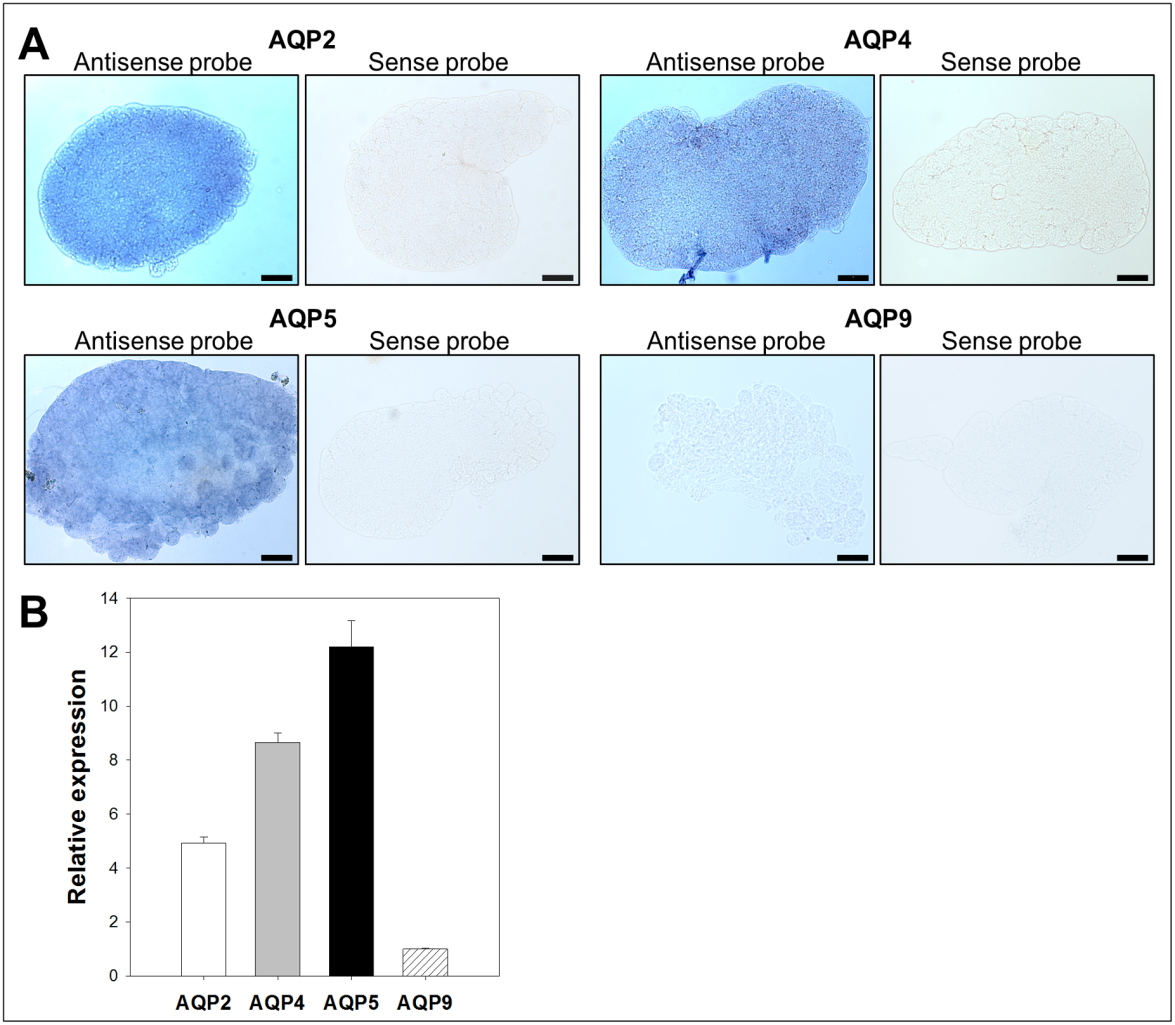
Expression analysis of *B. cockerelli* aquaporin candidates in bacteriocytes. A: *In situ* hybridization of candidate mRNAs with digoxigenin-labelled antisense and sense RNA probes (original magnification: ×20, bars = 50 µm). B: Relative gene expression levels of *B. cockerelli* aquaporin candidate genes normalized to the expression value of the RPS18 gene. Data points represent means ± SD of two independent experiments performed in duplicates.

**Figure 5:**
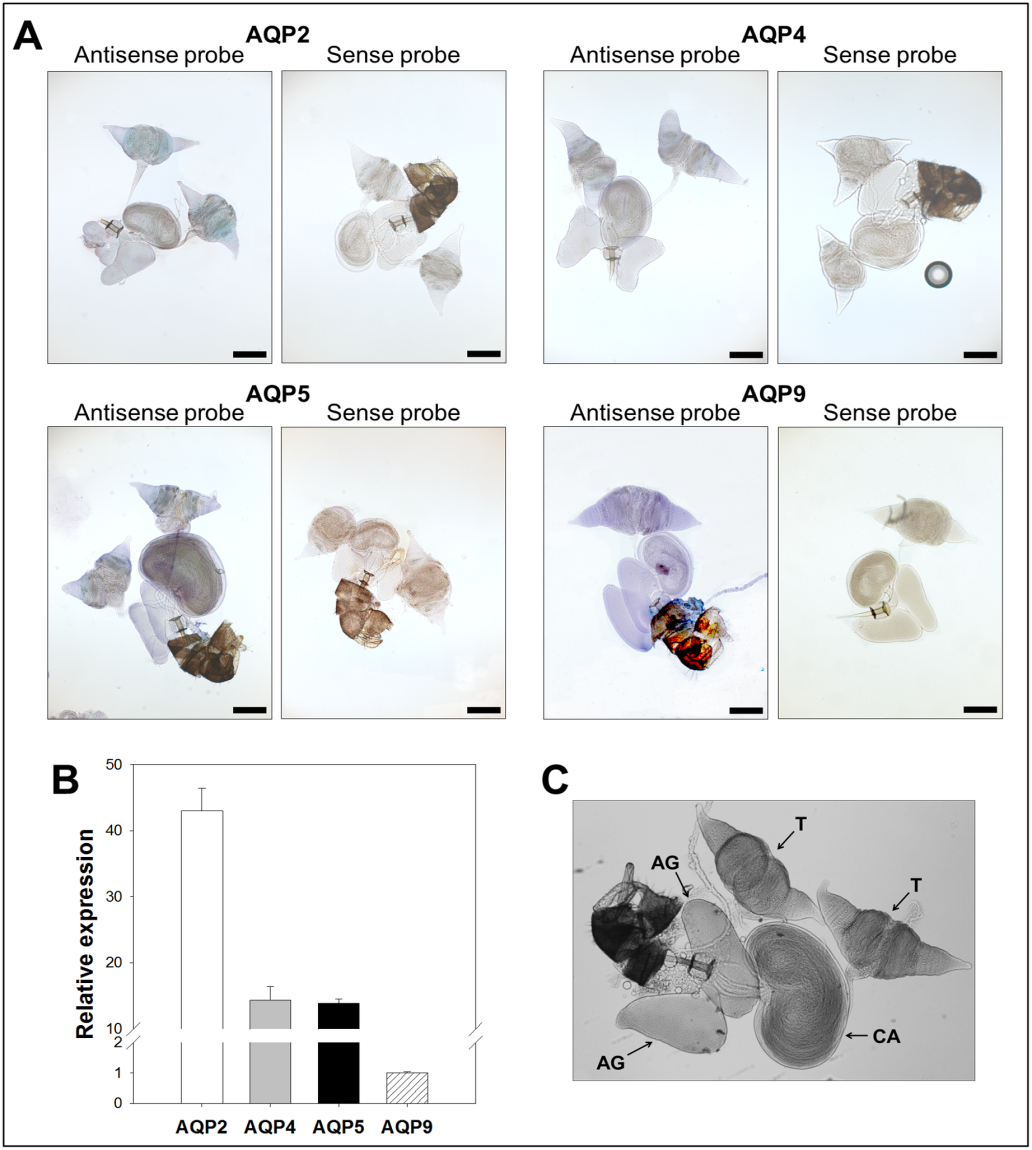
Expression analysis of *B. cockerelli* aquaporin candidates in male reproductive organs. A: *In situ* hybridization of candidate mRNAs with digoxigenin-labelled antisense and sense RNA probes (original magnification: ×5, bars = 200 µm). B: Relative gene expression levels of *B. cockerelli* aquaporin candidate genes normalized to the expression value of the RPS18 gene. Data points represent means ± SD of two independent experiments performed in duplicates. C. Light micrograph of *B. cockerelli* male reproductive organs showing characteristic structures. T: testes, AG: accessory glands, CA: scrotal capsule.

**Figure 6:**
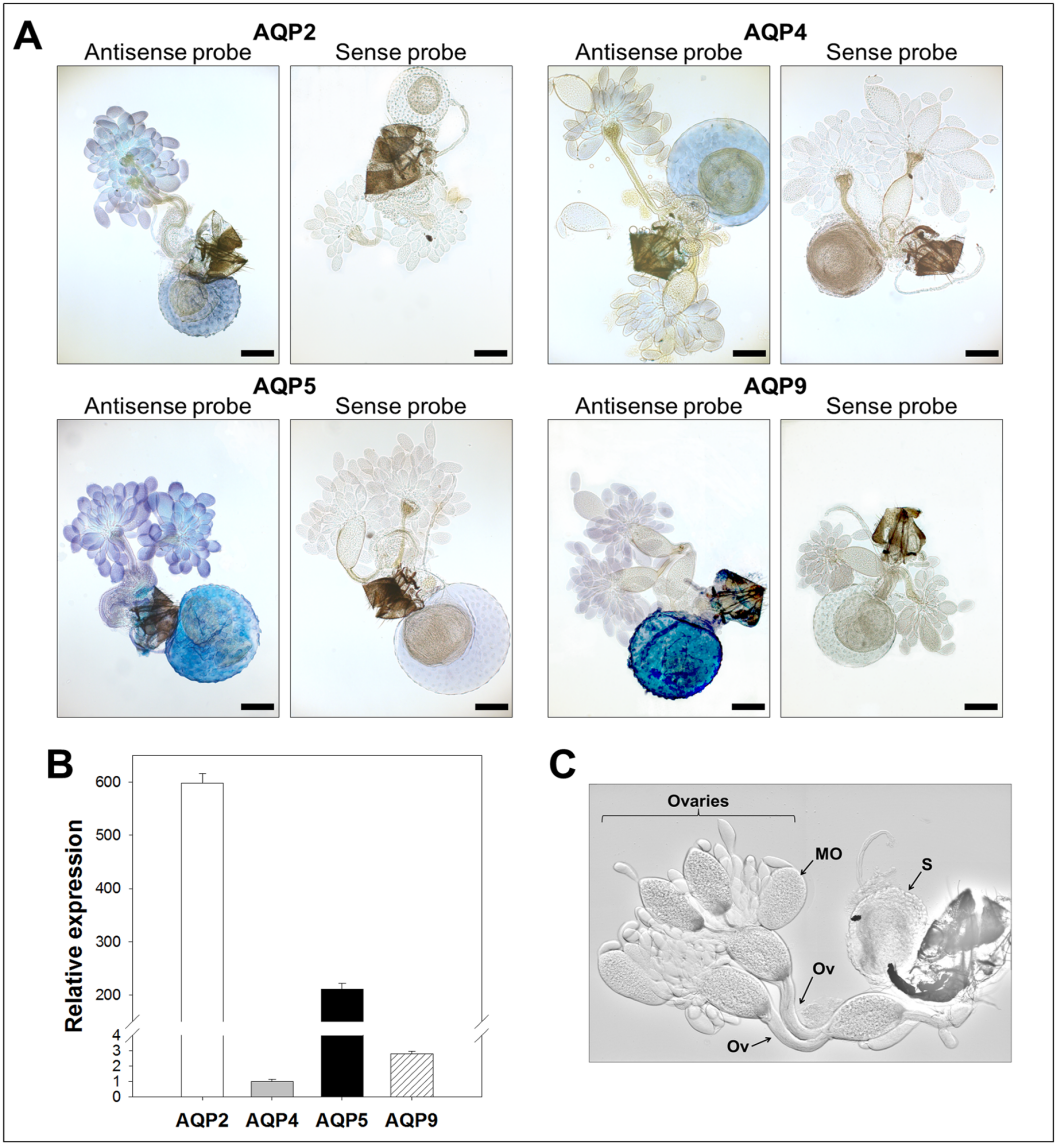
Expression analysis of *B. cockerelli* aquaporin candidates in female reproductive organs. A: *In situ* hybridization of candidate mRNAs with digoxigenin-labelled antisense and sense RNA probes (original magnification: ×5, bars = 200 µm). Of notice is the unspecific labelling of the spermatheca with BcAQP5-like sense probe. B: Relative gene expression levels of *B. cockerelli* aquaporin candidate genes normalized to the expression value of the RPS18 gene. Data points represent means ± SD of two independent experiments performed in duplicates. C. Light micrograph of *B. cockerelli* female reproductive organs showing characteristic structures. MO: mature oocyte, S: spermatheca, Ov: oviduct.

Since the candidates were identified from adult *B. cockerelli* cDNA libraries, their expression was analyzed by *in situ* hybridizations and confirmed using RT-qPCR (Figures 3–6) from adult dissected guts, bacteriocytes, female and male reproductive organs (negative controls using sense probes are shown in Figure 3–6).

Reverse transcription-qPCR analyses showed that all aquaporin candidate transcripts were expressed in the gut (Figure 3B) where BcAQP2-like transcript was detected at higher level than the other aquaporin candidates. Contrary to what is seen in other hemipteran’s aquaporins, we could not observe any particular pattern of expression within the gut for any candidate aquaporin (Figure 3A). Particularly, labeling of guts with BcAQP9-like probe was undetectable, which is in agreement with the low relative expression level obtained by RT-qPCR analyses.

In bacteriocytes, we found expression of all candidate genes by RT-qPCR (Figure 4B). As previously, BcAQP9-like was the candidate expressed at the lowest level. A homogenous labeling of BcAQP2-like, BcAQP4-like and BcAQP5-like transcripts was observed by *in situ* hybridization (Figure 4A). No signal was observed for BcAQP9-like transcript as expected based on the low relative expression level measured by RT-qPCR.

Expression of all aquaporin candidates was detected in the male reproductive tissues (Figure 5B). A high relative expression level was observed by RT-qPCR analyses for BcAQP2-like, while BcAQP9-like relative expression was the lowest. A localized labeling of BcAQP2-like, BcAQP4-like and BcAQP5-like was observed by *in situ* hybridization in the testes (Figure 5A).

In the female reproductive tissue, low relative expression levels of BcAQP9-like and BcAQP4-like were measured by RT-qPCR while high relative expression levels were measured for BcAQP5-like and BcAQP2-like (Figure 6B). For all candidates, labeling by *in situ* hybridization was observed in the ovaries (Figure 6A). Similarly, spermathecae were labeled with all probes (Figure 6A), however a low unspecific signal was observed with the BcAQP5-like sense probe (Figure 6A).

### Expression of *Bactericera cockerelli* aquaporins in different life stages

**Figure 7:**
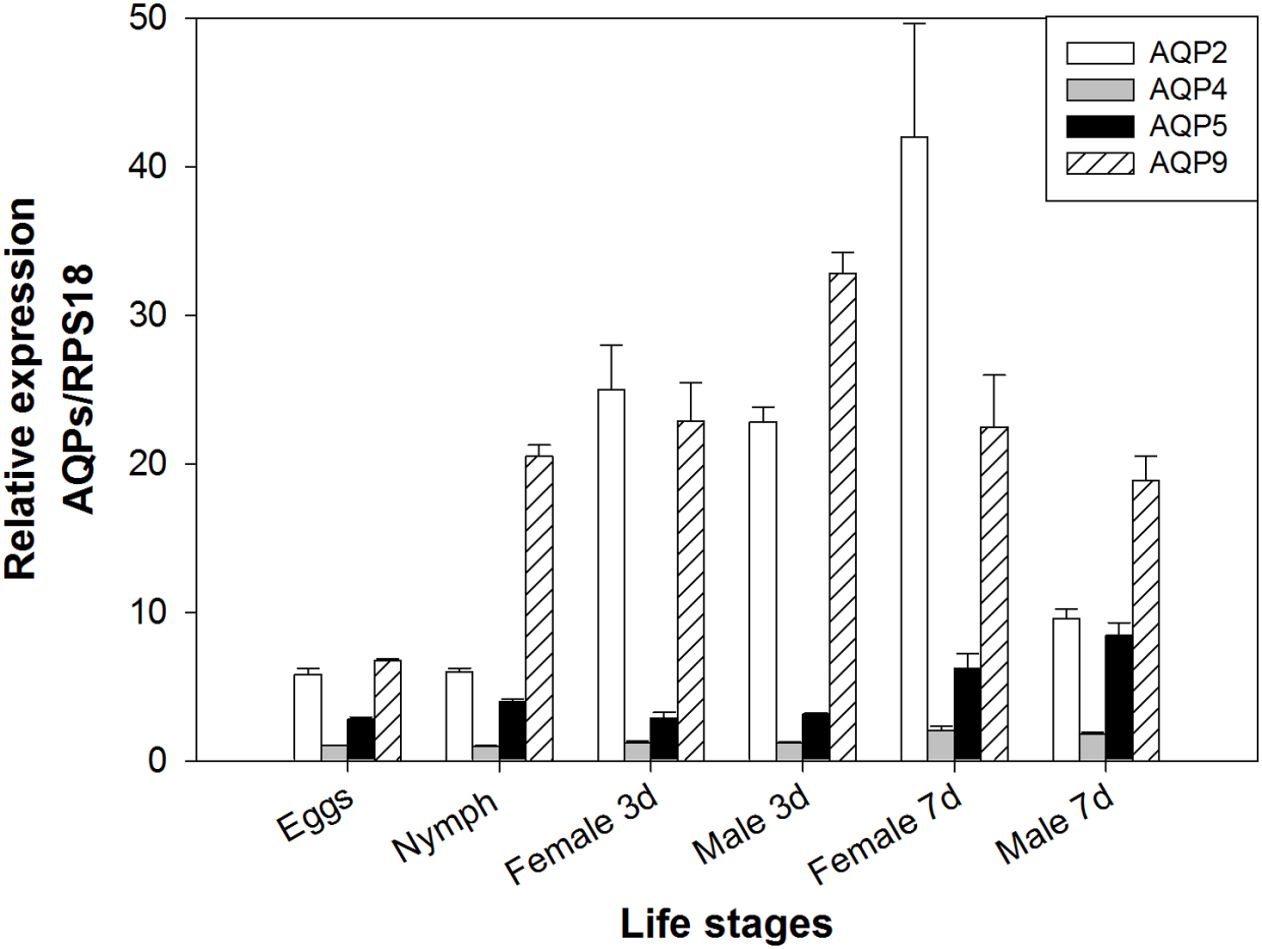
Expression analysis of *B. cockerelli* aquaporin candidates in different life stages. Relative gene expression levels of *B. cockerelli* aquaporin candidate genes normalized to the expression value of the RPS18 gene. Data points represent means ± SD of three independent experiments performed in duplicates.

Analysis of expression by RT-qPCR in different life stages showed that the four candidates were expressed in all tested life stages (Figure 7). In general, lower expression levels were measured in eggs than in the other evaluated life stages. A high relative expression level of BcAQP9-like was obtained using whole body cDNA as template. However, in expression analyses using cDNA from dissect tissues, this candidate showed low relative expression levels. Except in nymphs, a similar expression pattern was observed for BcAQP2-like and BcAQP9-like. BcAQP4-like and BcAQP5-like transcripts showed similar expression profiles throughout the insect development.

## Discussion

This study is the first global analysis of a phloem feeding insect to identify putative aquaporins. Aquaporins have been identified in animals, plants and microorganisms, but only recently important efforts have been aimed at increasing the knowledge of insect AQPs [7], [8], [21], [22], [23], [31], [33], [34], [35]. In spite of the potential importance of AQPs in phloem feeding hemipterans, no global report for any such species has been performed and only three reports of AQPs in phloem feeding insects exist [7], [8], [23]. High-throughput sequencing analyses have facilitated the identification of gene families in organisms without a sequenced genome. In this study, using transcriptomic datasets [24] we identified putative aquaporin genes in *B. cockerelli,* the vector of the pathogen ‘*Candidatus* Liberibacter solanacearum’, the causative agent of Zebra chip disease [36], [37], [38].

Four putative AQP candidates were identified: BcAQP2-like, BcAQP4-like, BcAQP5-like and BcAQP9-like. In phylogenetic analysis (Figure 2), only one of these candidates, BcAQP2-like, clustered within the aquaporin clade A, which comprises classical animal aquaporins (DRIPs, PRIPs and BIBs). This candidate clustered with PRIP proteins, an AQP group shown to transport water [35], [39], [40] or water and urea [41]. Two of the candidates, BcAQP4-like and BcAQP5-like, clustered within clade B, previously described as an insect-specific clade. The long C-loop found in these two *B. cockerelli* AQP genes might suggest an aquaglyceroporin function [31]. Moreover, several members of clade B have been validated as functionally active aquaglyceroporins [23], [34]. The last candidate, BcAQP9-like also clustered in clade B, however this candidate appeared to be more divergent. As more insect aquaporin sequences are obtained, a better clade support will be achieved thus possibly modifying the clade shape.

Presently, three aquaporins from phloem feeding insects have been characterized functionally. Two of these candidates, the DRIP-like ApAQP1 and BtAQP1 were shown to be expressed within the insect gut, where the proximal and distal regions are in close contact [7], [8]. None of the *B. cockerelli* candidate genes showed similar expression localization (Figure 3A). The four *B. cockerelli* aquaporin candidates were found expressed in the gut as shown in Figure 3B. However, none of them appeared restricted to the filter chamber. It is possible that the expression level and localization of these genes might change in response to active feeding. For instance, a dynamic expression level after feeding of two *R. prolixus* aquaporin genes, RhoprAQP1 and RhoprMIP-A, was reported [22]. The insects used in our analyses were randomly picked from the colonies independently of their behavior at the time of collection (feeding, resting, jumping, mating, etc). Future studies could focus on gene and protein expression and localization in *B. cockerelli* before, during and after feeding.

The third phloem feeding hemipteran aquaporin, the aquaglyceroporin ApAQP2, was found expressed in the fat body and bacteriocyte [23]. Bacteriocytes are special cells that harbor the primary endosymbiont of hemipterans. All *B. cockerelli* candidate genes were found expressed within the bacteriocyte. Interestingly, bacteriocytes were the only tested organs in which we obtained a higher relative expression of BcAQP4-like and BcAQP5-like than BcAQP2-like, the most expressed AQP candidate in all dissected organs (Figure 4). Based on sequence analysis of the candidate aquaporins and their similarity to other insect aquaporins such as ApAQP2, BcAQP4-like and BcAQP5-like could potentially function as aquaglyceroporins. ApAQP2 is believed to be involved in substrate transport to support the symbiosis with the aphid primary endosymbiont *Buchnera aphidicola* [23]. Therefore, it is possible that BcAQP4-like and BcAQP5-like might be involved in supporting the symbiosis with the psyllid primary endosymbiont *Carsonella ruddii*. Furthermore, the expression of these two *B. cockerelli* genes remained relatively constant across all insect life stages, with a slight increase in older adults (Figure 7).

The candidate BcAQP9-like showed no similarity to any insect aquaporin already characterized. Its relative expression within all adult organs assayed by RT-qPCR was low (Figures 3B–6B), however, it was one of the candidates with higher relative expression in all life stages when whole insects were tested (Figure 7). Thus, BcAQP9-like could be expressed in organs or tissues not assayed in this study. For example, this candidate might be expressed in salivary glands where it might be involved in saliva secretion, in muscles, in fat body, or in the eyes.

In summary, this study is the first global attempt to identify aquaporin candidates in a phloem feeding insect. The genome of the psyllid *Diaphorina citri* is being sequenced; its annotation will help shed light on the repertoire of aquaporin genes found in these hemipterans. Preliminary analyses such as the annotation of *A. pisum* genome and this study point that hemipterans might have a reduced repertoire of aquaporin genes compared to other insects. Aquaporins involved in sustaining the primary endosymbiont might be primary candidates to disrupt this interaction.

## Supporting Information

Figure S1 **Nucleotide and** ***in silico*** **deduced amino acid sequences of BcAQP2-like.** The NPA motifs are boxed and the predicted transmembrane regions are shaded in grey.
(TIF)

Figure S2 **Nucleotide and** ***in silico*** **deduced amino acid sequences of BcAQP4-like.** The NPA motifs (NPS and NPA) are boxed and the predicted transmembrane regions are shaded in grey.
(TIF)

Figure S3 **Nucleotide and** ***in silico*** **deduced amino acid sequences of BcAQP5-like.** The NPA motifs (NPT and NPA) are boxed and the predicted transmembrane regions are shaded in grey.
(TIF)

Figure S4 **Nucleotide and** ***in silico*** **deduced amino acid sequences of BcAQP9-like.** The NPA motifs are boxed and the predicted transmembrane regions are shaded in grey.
(TIF)
