# Supplementary figures and images for: Identification and Expression Analysis of Aquaporins in the Potato Psyllid, Bactericera cockerelli
Source: PLoS One. 2014 Oct 29;9(10):e111745. doi: 10.1371/journal.pone.0111745 (PMC4213062; doi:10.1371/journal.pone.0111745)

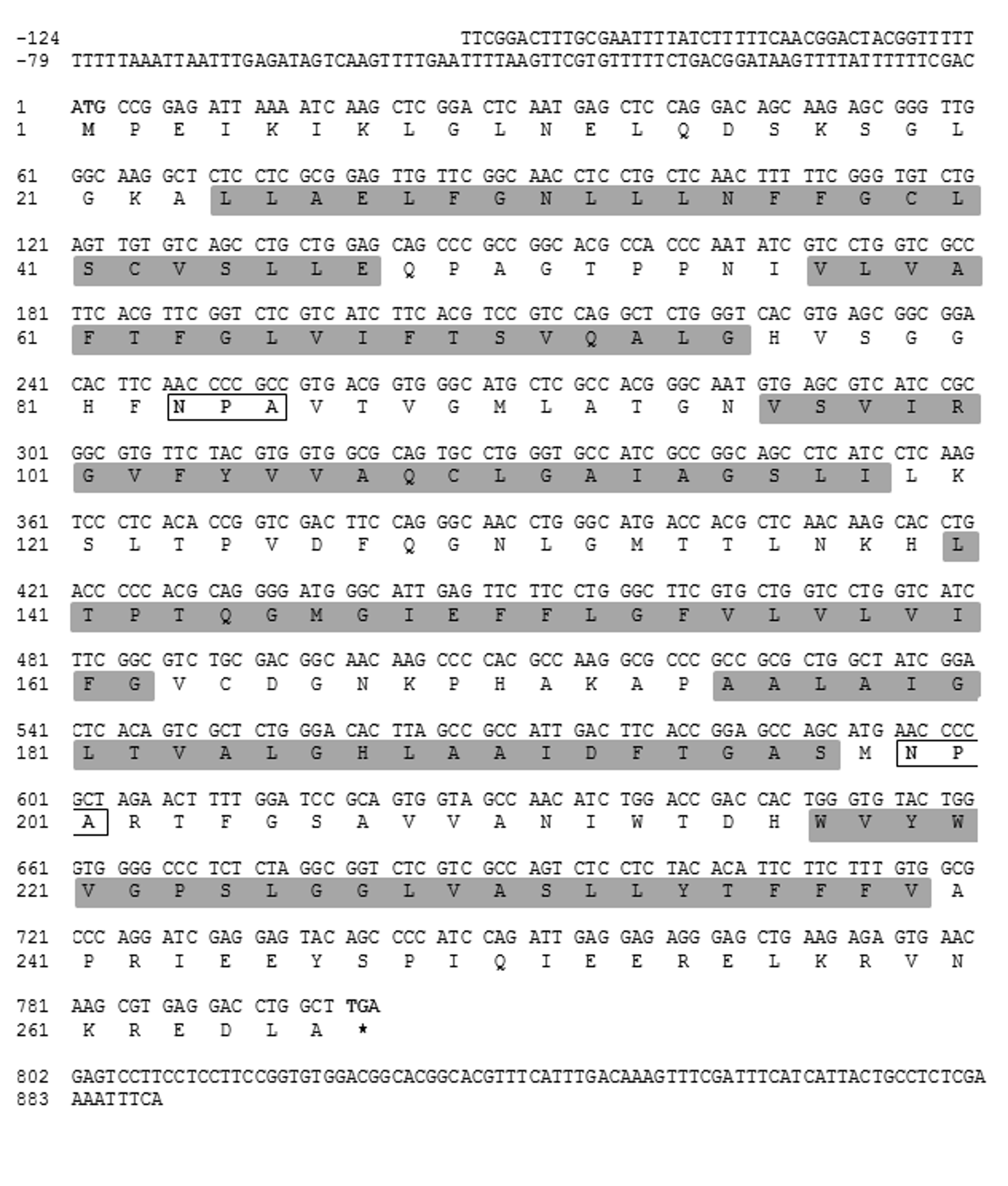

Supplement: Figure S1 — Nucleotide and in silico deduced amino acid sequences of BcAQP2-like. The NPA motifs are boxed and the predicted transmembrane regions are shaded in grey. (TIF) [file pone.0111745.s001.tif]

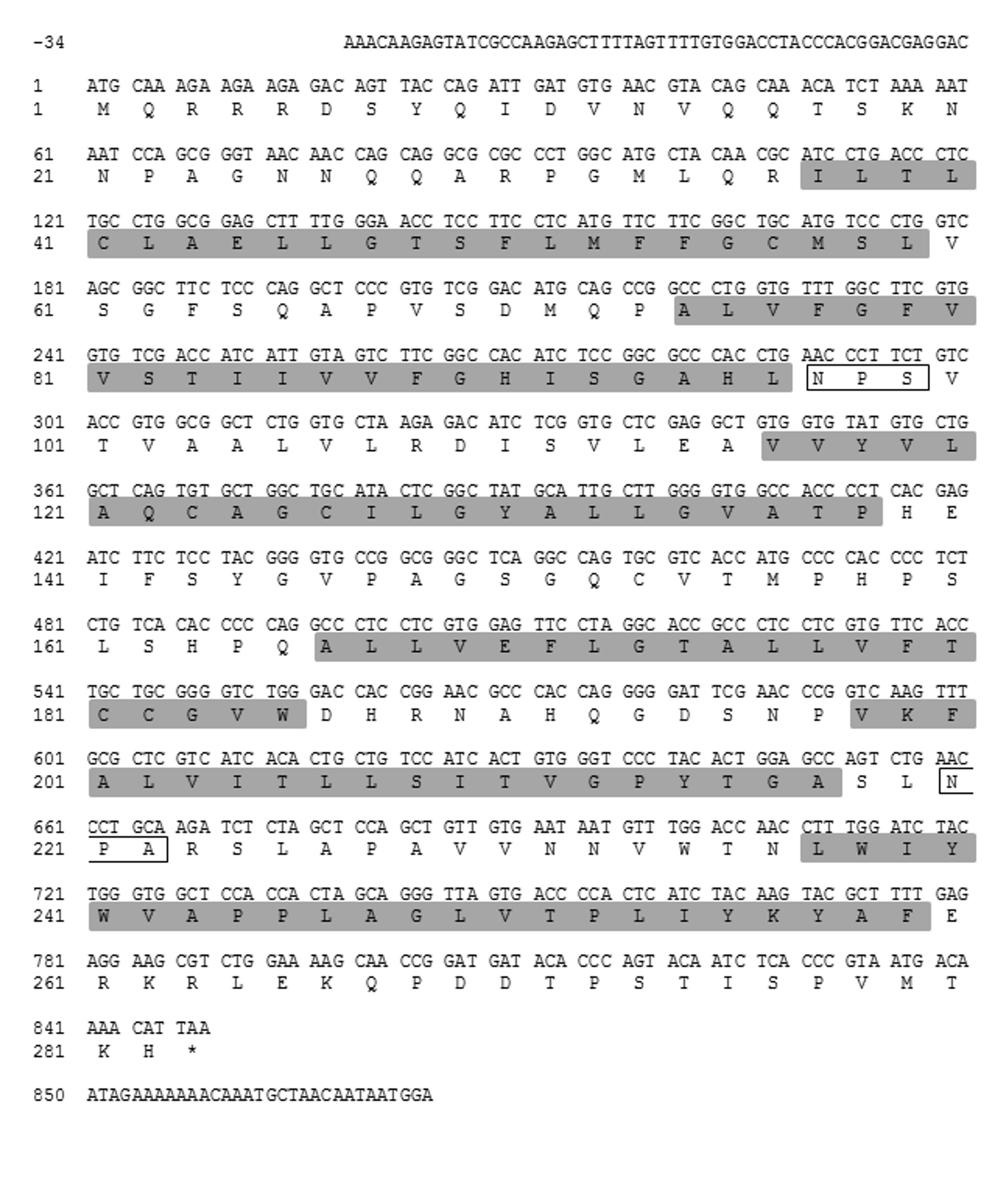

Supplement: Figure S2 — Nucleotide and in silico deduced amino acid sequences of BcAQP4-like. The NPA motifs (NPS and NPA) are boxed and the predicted transmembrane regions are shaded in grey. (TIF) [file pone.0111745.s002.tif]

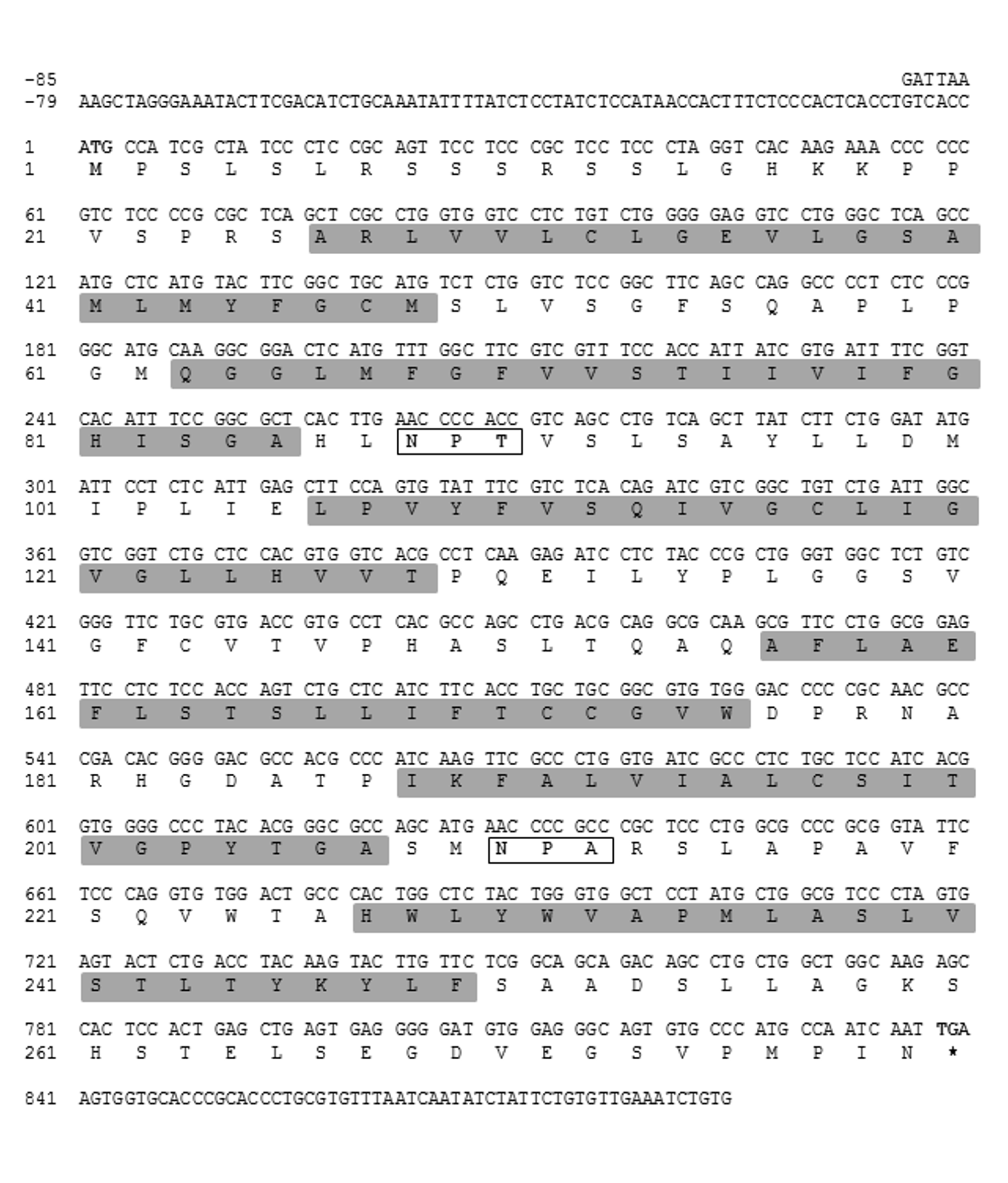

Supplement: Figure S3 — Nucleotide and in silico deduced amino acid sequences of BcAQP5-like. The NPA motifs (NPT and NPA) are boxed and the predicted transmembrane regions are shaded in grey. (TIF) [file pone.0111745.s003.tif]

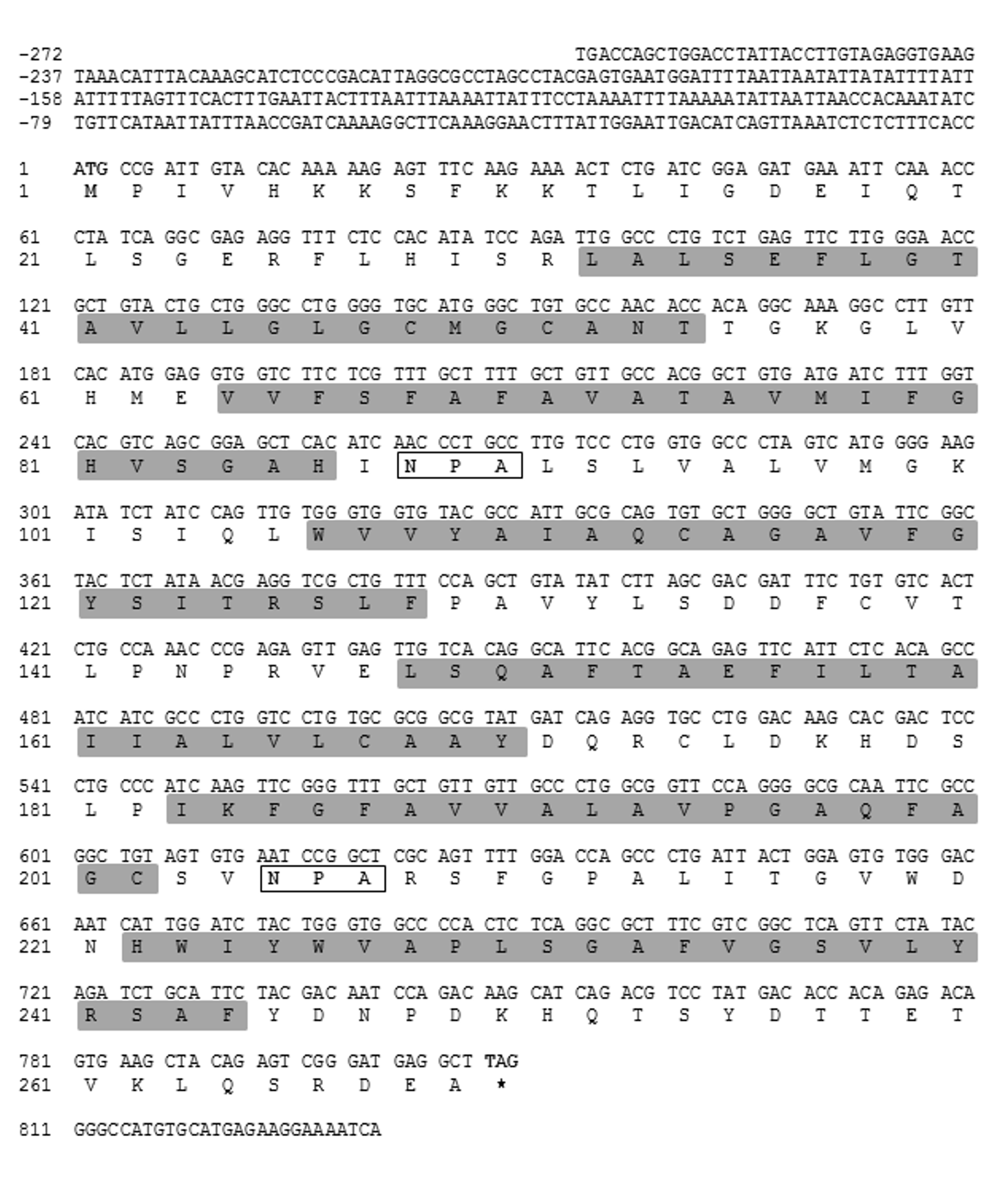

Supplement: Figure S4 — Nucleotide and in silico deduced amino acid sequences of BcAQP9-like. The NPA motifs are boxed and the predicted transmembrane regions are shaded in grey. (TIF) [file pone.0111745.s004.tif]
